# Add-on treatment with *N*-acetylcysteine for bipolar depression: a 24-week randomized double-blind parallel group placebo-controlled multicentre trial (NACOS-study protocol)

**DOI:** 10.1186/s40345-018-0117-9

**Published:** 2018-04-05

**Authors:** Pernille Kempel Ellegaard, Rasmus Wentzer Licht, Henrik Enghusen Poulsen, René Ernst Nielsen, Michael Berk, Olivia May Dean, Mohammadreza Mohebbi, Connie Thuroee Nielsen

**Affiliations:** 1 Institute of Regional Health Services Research, Faculty of Health Sciences, University of Southern Denmark, Odense, Denmark; 2 Research Unit, Mental Health Service Esbjerg, Gl. Vardevej 101, 6715 Esbjerg N, The Region of Southern Denmark Denmark; 3 OPEN, Odense Patient Data Explorative Network, Odense University Hospital, Institute of Clinical Research, University of Southern Denmark, Odense, Denmark; 4 Unit for Psychiatric Research, Psychiatry, Aalborg University Hospital, Aalborg, Denmark; 5 Department of Clinical Medicine, Faculty of Medicine, Aalborg University, Aalborg, Denmark; 6 Clinical Pharmacology, Bispebjerg Frederiksberg Hospital, Copenhagen, Denmark; 7 Institute of Clinical Medicine, Faculty of Health and Medical Sciences, University of Copenhagen, Copenhagen, Denmark; 8 IMPACT Strategic Research Centre, School of Medicine, Barwon Health, Deakin University, Geelong, Australia; 9 Florey Institute for Neuroscience and Mental Health, University of Melbourne, Parkville, Australia; 10 Department of Psychiatry, University of Melbourne, Royal Melbourne Hospital, Parkville, Australia; 11 Biostatistics Unit, Faculty of Health, Deakin University, Geelong, Australia; 12 Mental Health Service Vejle, Odense, The Region of Southern Denmark Denmark

**Keywords:** Bipolar disorder, Depression, Acetylcysteine, Oxidative stress, Inflammation, Urine, Blood, Treatment, Psychiatry, Genetics

## Abstract

**Background:**

Oxidative stress and inflammation may be involved in the development and progression of mood disorders, including bipolar disorder. Currently, there is a scarcity of useful treatment options for bipolar depressive episodes, especially compared with the efficacy of treatment for acute mania. *N*-Acetylcysteine (NAC) has been explored for psychiatric disorders for some time given its antioxidant and anti-inflammatory properties. The current trial aims at testing the clinical effects of adjunctive NAC treatment (compared to placebo) for bipolar depression. We will also explore the biological effects of NAC in this context. We hypothesize that adjunctive NAC treatment will reduce symptoms of depression, which will be reflected by changes in selected markers of oxidative stress.

**Methods and analysis:**

In the study, we will include adults diagnosed with bipolar disorder, in a currently depressive episode. Participants will undertake a 20-week, adjunctive, randomized, double-blinded, parallel group placebo-controlled trial comparing 3 grams of adjunctive NAC daily with placebo. The primary outcome is the mean change over time from baseline to end of study on the Montgomery–Asberg Depression Rating Scale (MADRS). Among the secondary outcomes are mean changes from baseline to end of study on the Bech-Rafaelsen Melancholia Scale (MES), the Young Mania Rating Scale (YMRS), the WHO-Five Well-being Index (WHO-5), the Global Assessment of Functioning scale (GAF-F), the Global Assessment of Symptoms scale (GAF-S) and the Clinical Global Impression-Severity scale (CGI-S). The potential effects on oxidative stress by NAC treatment will be measured through urine and blood samples. DNA will be examined for potential polymorphisms related to oxidative defences.

*Trial registration*: Registered at The European Clinical Trials Database, ClinicalTrials.gov: NCT02294591 and The Danish Data Protection Agency: 2008-58-0035.

## Background

Bipolar disorder is generally characterized by recurrent episodes of mania (or hypomania) and major depression (Goodwin and Jamison 2007), with depressive phases being the dominant phase in terms of overall duration (Judd and Akiskal 2003).

In recent treatment guidelines for bipolar disorder, combinations of pharmacotherapy and psychotherapeutical treatments such as psychoeducation and individual or group-based psychotherapy are generally recommended (Fountoulakis et al. 2017). Regarding pharmacotherapy, combined use of various drugs is often beneficial in terms of both acute treatment response and for maintenance (Geddes et al. 2016). However, despite all efforts, treatment outcome is often inadequate, necessitating the development of new, more effective treatments. There a scarcity of effective treatments for the acute treatment of bipolar depression, in particular (Grunze et al. 2010).

Studies have indicated that oxidative stress and neuroinflammation seem to be linked to the pathophysiology of bipolar disorder (Andreazza et al. 2008; Berk et al. 2011). Recent studies have reported increased levels of oxidative stress markers in blood and urine samples from patients suffering from bipolar disorder (Andreazza et al. 2007, 2008; Munkholm et al. 2015; Jacoby et al. 2016). Furthermore, there is evidence that the level of oxidative stress may be a state marker in bipolar disorder (Munkholm et al. 2015; Fernandes et al. 2011; Polyakova et al. 2015).

The brain is thought to be especially vulnerable to oxidative stress due to its’ high metabolic demands and its’ limited capacity of defences such as glutathione (GSH), which is the central antioxidant in all tissues (Ballatori et al. 2009). Bipolar disorder is associated with decreased GSH levels, resulting in increased oxidative stress (Andreazza et al. 2008; Rosa et al. 2014; Brown et al. 2014; Soeiro-de-Souza et al. 2013). *N*-Acetylcysteine (NAC), the acetyl derivative of the amino acid cysteine, improves the l-cysteine supply, which increases GSH levels in the brain (Choy et al. 2010; Dean et al. 2009). Accordingly, NAC treatment appears to decrease oxidative stress and inflammation (Dean et al. 2011). Two previous clinical trials on NAC for bipolar disorder have shown significant effects on depressive symptoms (Berk et al. 2008, 2011).

### Study rationale

Given the paucity of effective pharmacotherapy for bipolar depression, it seems warranted to test whether the previous positive findings of NAC for bipolar depression can be replicated in a Danish study sample. Furthermore, we wanted to go further than these studies by exploring the antioxidant effects of NAC in the context of the clinical trial. Finally, it would be useful for the development of new pharmacotherapies in general to test whether the potential antidepressant efficacy of NAC is linked to its antioxidant effects. In addition to the latter point, finding a relationship between the level of oxidative stress at baseline and the efficacy of NAC would be a step towards the goal of precision psychiatry (Fernandes et al. 2017).

### Aims and objectives

The primary aim is to investigate the effects of adjunctive NAC treatment on depressive symptoms in participants diagnosed with bipolar depression. The secondary aims are to measure the biological effect of NAC treatment based on markers of oxidative stress in urine samples.

The study objectives are following:

#### Process aim 1

Assessing baseline adjusted between-group mean change between NAC and placebo group from baseline up till week 20 on The Montgomery–Asberg Depression Rating Scale score (MADRS) (Montgomery and Åsberg 1979).

#### Process aim 2

Assessing baseline adjusted between-group mean difference between NAC and placebo group from baseline up till week 20 on; the Bech-Rafaelsen Melancholia Scale (MES) (Bech 2002), the Young Mania Rating Scale (YMRS) (Young et al. 1978), the WHO-Five Well-being Index (WHO-5) (Staehr 1998), the Global Assessment of Functioning scale (GAF-F), The Global Assessment of Symptoms scale (GAF-S) (Jones et al. 1995) and the Clinical Global Impression-Severity scale (CGI-S) (Guy WNIoMHPRBECDEP 1976).

#### Process aim 3

Assessing baseline adjusted between-group mean difference between NAC and placebo group from baseline up till week 20 in urine samples to explore biological correlation in DNA and RNA damage by oxidative stress.

#### Process aim 4

Assessing baseline adjusted between-group mean difference between NAC and placebo group from baseline up till week 24 on MADRS, MES, YMRS, WHO-5, GAF-F, GAF-S, CGI-S and urinary markers.

#### Process aim 5

Assessing baseline urinary markers to explore correlation in DNA and RNA damage by oxidative stress and the degree of depression severity.

Additionally, blood samples and DNA are collected for further explorations towards detecting potential genetic polymorphisms related to oxidative defences, but are currently stored.

## Methods

### Trial design

This is a 20-week randomized, double-blind, parallel group, placebo-controlled, multicentre trial, with a 4-weeks follow up after study treatment discontinuation. The protocol is developed in accordance with the Standard Protocol Items: Recommendations for Intervention Trials (SPIRIT) 2013 Guidelines (Chan et al. 2013). The study will follow the Consolidated Standards of Reporting Trials (CONSORT) guidelines to describe, report and publish the results (Schulz et al. 2010).

### Visits and telephone calls

**Fig. 1:**
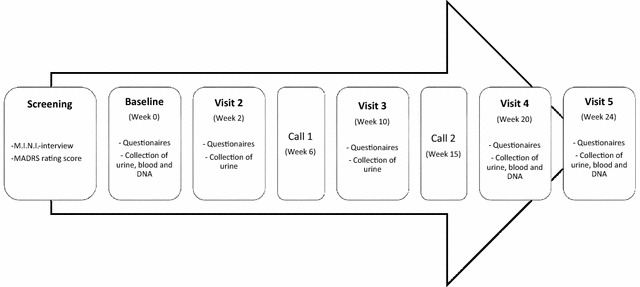
Timeline with outcomes measures. *M.I.N.I-interview* Mini International Neuropsychiatric Interview, *MADRS* Montgomery–Asberg Depression Rating Scale

The study visits (outlined in Fig. 1) are planned to be at week 2, 10, 20 and 24 (± 7 days for each visit). Furthermore, participants will be contacted via telephone 6 and 15 weeks after inclusion (± 7 days). Telephone calls are conducted with participants to support adherence and provide an opportunity for participants to discuss any issues with the study (including adverse events).

### Participant selection

The study participants will be recruited amongst in-and out patients from the Mental Health Services from three study centres covering three geographical areas (three cities in centre one and two, and one city in centre three). We plan to recruit participants over a study period of 2 years.

Participants assess for eligibility will be screened according to the inclusion and exclusion criteria.

### Participant eligibility

#### Inclusion criteria

Participants will be included if they meet all of the following criteria:

- 18–64 years of age at baseline (inclusive).
- A diagnosis of bipolar disorder type I or II, current episode of depression according to Diagnostic and Statistical Manual of Mental Disorders 5 (DSM-V) (American Psychiatric Association 2000) (evaluated by using the M.I.N.I (International Neuropsychiatric Interview), version 5.0 in Danish (Sheehan et al. 1998).
- At least one documented affective episode within the last 6 months within the index period (depressive, manic or mixed), as judged by the principal investigator (according to the medical journal, and clinical interview based on the M.I.N.I
- Have had symptoms of depression for at least 4 weeks up till screening, and a Montgomery–Asberg Depression Rating Scale (MADRS) score of ≥ 18 (mild depression or more) at that time.
- Have given full oral and written informed consent.

Female participants should present a negative pregnant test at baseline and will be informed to use effective contraception throughout the study period.

#### Exclusion criteria

Participants will be excluded if they meet any of the following criteria:

- Have a daily intake of more than 500 mg NAC, 200 μM selenium, or 500 IU of vitamin E at screening.
- Have received electroconvulsive therapy (ECT) within the last 4 weeks prior to screening.
- Have epilepsy, chronic asthma or allergy towards NAC, as judged by investigator.
- Involuntary admitted, detainment or treated according to the statutes defined in the Mental Health Act.
- Cannot speak or understand the Danish language.
- Females planning pregnancy or breastfeeding during the timeframe of the study.

#### Discontinuation criteria

Participants will discontinue the study under the following circumstances:

- Withdrawal of consent.
- Discontinues study treatment for 7 or more consecutive days.
- Does not comply with the defined study visits (visits within 7 days prior or after the computer-generated date).
- Becomes pregnant, initiates breastfeeding, or wishes to become pregnant during the study period.
- Becomes hypersensitive to histamine, as judged by investigator.
- Develops serious adverse reaction/event (SAR/SAE) i.e. suspected to be associated with the study treatment, as judged by an investigator.
- Stops using effective contraception.
- Changes diagnosis during the study, and the primary diagnosis is no longer bipolar affective disorder.
- Involuntary admitted, detainment or treated according to the statutes defined in the Mental Health Act.
- Becomes increasingly suicidal during the study as judged by investigator.

### Randomization

Participants will be randomized to NAC or placebo treatment by a computer-based random eight block number generator. The study participants will be randomised sequentially by the order they are enrolled into the trial. The randomization assignments will be hidden from the participants and the study investigators and raters. An independent pharmacy, Glostrup Pharmacy, will perform the randomization process, provide the randomization codes and the labelling of the tablet containers. The Coordinating Investigator (PKE) and Sponsor/Principal Investigator (CTN) will enrol and assign participants from two of the study centres, and the second Principal Investigator (REN) and a project nurse (HOE) will enrol and assign participants from the latter study centre.

### Blinding procedure

The study medication is blinded to both study participants and the investigators. If un-blinding of one or more study participants is required, Glostrup Pharmacy hold the randomization list and can be contacted 24 h a day. Furthermore, the investigators have a sealed envelope with the randomization list, if there is a need for un-blinding of any participants. To minimize the risk of participants comparing experiences, the study visits will be arranged staggered throughout the day and always separately. When all study participants have completed the trial, the study medication will be un-blinded.

### Study treatment

Participants will be randomized to receive either NAC (National Center for Biotechnology Information 2017) or placebo in addition to their current pharmacotherapy and psychotherapy.

The study treatment powder (NAC and placebo) are imported and manufactured at Glostrup Pharmacy, which has manufacturing authorization, and all medication used is manufactured in accordance with good manufacturing practice (GMP) and good distribution practice (GDP).

The study medication is divided into nine, white containers containing 100 tablets each. The study medication is distributed twice during the 20 weeks, and receive 9 containers in total.

Participants will take three tablets containing 500 mg each, twice daily (3 g daily) for 20 weeks. NAC and placebo tablets have a similar appearance and have an added lemon odour, in order to camouflage the sulphur-containing odour of NAC.

ECT is not permitted 4 weeks prior to inclusion, but is allowed during the trial. Treating clinicians are allowed to change both psychopharmacy and psychotherapy during the trial at their discretion.

### Treatment adherence

To enhance adherence, the participants will be offered a text message with two daily reminder messages on taking the study medication. The participants will be encouraged to return all remaining trial medication at the end of study participation. The returned tablet containers and the tables will all be counted and documented.

### Outcome measures

The clinical and demographic data is obtained by the investigators through patient interviews and from hospital records. The biological data is obtained through laboratory analyses of blood and urine samples. An overview of the data collection process is displayed in Table 1.

**Table 1:** Overview of all measurements, instruments and time points

Variables	Sources	Study period					
		Enrolment	Intervention				Follow-up
		Screening	Baseline (week 0)	Visit 2 (week 2)	Visit 3 (week 10)	Visit 4 (week 20)	Visit 5 (week 24)
MADRS	Rater	X		X	X	X	X
MES, WHO-5, GAF-S, GAF-F, CGI-S YMRS	Rater/self-reported		X	X	X	X	X
M.I.N.I.-Interview	Rater	X					
Demographic (age, gender, height, inclusion site, etc.)	Self-reported		X				
Weight and BMI	Rater		X	X	X	X	X
Personal and demographic details (income source, education, relationship etc.)	Self-reported		X				
Family mental history	Self-reported		X				
Lifestyle information (smoking status, alcohol consumption and substance abuse)	Self-reported		X	X	X	X	X
Disorder information (no. of: episodes of depression, hypomania/mania, neutral mood, ECT, hospital admissions, suicidal thoughts, suicide attempts, etc.)	Self-reported/hospital record		X				
Medical conditions (Doctor diagnosed)	Self-reported		X				
Pharmacological treatment	Hospital record		X	X	X	X	X
Psychological treatment	Self-reported		X	X	X	X	X
Adverse events/reactions	Self-reported		X	X	X	X	X
Biography	Self-reported			X			
Urine samples	Laboratory		X	X	X	X	X
Blood samples	Laboratory		X			X	X
DNA	Laboratory		X			X	X

*Screening* pre-intervention, *Baseline* pre-intervention (week 0), *Visit 2* week 2, *Visit 3* week 10, *Visit 4* post-intervention (week 20), *Visit 5* follow-up (week 24), *Rater* coordinating investigator/project nurse, *MADRS* Montgomery-Åsberg Depression Rating Scale score, *MES* Bech-Rafaelsen Melancholia Scale, *WHO-5* World Health Organization well-being index, *GAF-S* The Global Assessment of Symptoms scale, *GAF-F* The Global Assessment of Functioning scale, *YMRS* Young Mania Rating Scale (YMRS), *CGI-S* The Clinical Global Impression-Severity scale (CGI-I), *M.I.N.I-interview* Mini International Neuropsychiatric Interview, *ECT* electroconvulsive therapy

#### Clinical outcome measures

The clinical measurements include scales of depressive symptoms (MADRS and MES), manic symptoms (YMRS), quality of life (WHO-5), symptoms- and social functioning level (GAF-S and GAF-F) and global clinical impression (CGI-S). All rating scales used in the study are assessed and validated in Danish.

#### Biological outcome measures

The biological data collected from the study participants will include urine and blood samples as well as DNA. The biological data will be applied to investigate markers of oxidative stress and inflammation. All biological materials (urine, blood and DNA) will be collected from not-fasting participants. The urine and blood samples are transferred to tubes, and all three biological materials are stored in hospital freezers until analysis.

### Serious adverse reactions/events and adverse reactions/events

SARs, SAEs, Adverse Events (AE) and Adverse Reactions (AR) are defined according to good clinical practice (GCP), and assessed at each visit. Participants who discontinue study participation due to SAEs or SARs will be followed until the SAE/SAR has disappeared or the SAE/SAR is stable. Participants will be contacted with a few days after SAE/SAR and again after 4 weeks.

### Data management

The trial is conducted in accordance with good clinical practice guidelines and Declaration of Helsinki, and will be monitoring by the GCP-unit at Odense University Hospital, Denmark. The study is approved before study initiation by the Regional Scientific Ethical Committees for Southern Denmark: 35664-20120177, the Danish Medicines Agency: 2012-004483-22, and Danish Data Protection Agency: 2008-58-0035. The trial is registered at ClinicalTrials.Gov: NCT02294591 and the European Clinical Trials Database, EudraCT: 2012-004483-22.

All data in the CRFs (Case Report Forms) are double entered in the database program *Research Electronic Data Capture* (REDCap) (Harris et al. 2009) by coordinating investigator (PKE).

### Sample size and feasibility of recruitment

The sample size estimation was based on prior data from a similar clinical trial showing mean (SD) in MADRS score (from baseline to end of the study (week 24)) was decreased by around 10.0 ± 4.3 and increased by around 0.9 ± 2.6 (in respectively the NAC and the placebo group (Berk et al. 2008). Our sample size was calculated using a two-tailed independent sample t-test with 80% power to detect baseline-adjusted between-group mean difference at week 20, and a two-sided alpha-level of 0.05. To detect a between-group mean difference of change from baseline of at least 8 ± 10.0, 56 participants in total were required. Due to the expectation of a 40% drop-out rate we aim to recruit an overall of 78 study participants. To achieve an adequate number of participants, all in- and out-patients from the Mental Health Service included in the study having a bipolar disorder or suspect to have this disorder, are offered to participate.

### Statistical analysis plan

A statistical significant difference in mean change on MADRS-score, between study groups, from baseline to end of study, is the primary outcome measure of the study. The remaining clinical and biological measures are secondary outcome measures and will be analysed using the same approach as the primary outcome. To investigate the difference in response and remission rates between study treatments at week 20 and 24, logistic regression models will be applied to estimate the odds ratios. The definition of a “responder” is a person which symptoms of depression has dropped 50% in MADRS-score from baseline to week 20 or 24, and “remitters” are defined as MADRS score < 10 (Hawley et al. 2002) at week 20 or 24. Data analysis will be conducted by the authors and a statistician. Between-group comparisons will be performed by applying Student t-tests, Mann–Whitney U tests, Chi square tests or Fishers exact test, whichever is most appropriate. The primary analysis for primary and secondary outcomes will be based on an Intention-To-Treat (ITT) analysis comprise all study participants completed the baseline measurements. A generalized estimating equation (GEE) (Feng et al. 2001) approach will be taken, and the likelihood-based linear mixed effects repeated measures (LMERM) analyses (Mallinckrodt et al. 2001; Laird and Ware 1982) is the primary analysis. The LMERM-analysis includes the fixed variable *study treatment*, *visits* as factor variable, and *treatment*-*by*-*visits* interaction (intervention impact). The LMERM includes all available data at each time point and is the preferred methods of analysing clinical trial data in psychiatry due to the fact that likelihood approach deals with missing data in follow-ups assuming missingness is at random. Planned comparisons will be done with the LMERM models to determine between-group differences in score change from baseline to week 2, week 10, week 20 and week 24. Additional per-protocol analysis includes participants who completed the study intervention. Sensitivity analysis will evaluate missing at random assumptions for missing follow-up data. Missing data will be imputed assuming Missing Not At Random (MNAR) in intervention, control and both arms respectively and robustness of finding under different MNAR assumptions will be examined.

Additionally, effect sizes will be reported using Cohen’s guidelines (Cohen 1988). Dichotomous data will be presented as proportions, with 95% confidence interval, and reporting Chi square of Fishers’s exact p-value where appropriate. Likelihood based generalized linear mixed repeated measures approach (GLMRM) with logit link will be used to compare dichotomous outcomes.

All tests of treatment effects will be conducted using a two-sided alpha level of 0.05. The statistical analysis will be conducted utilizing the statistical software program STATA IC, version 15 (StataCorp. Stata Statistical Software: Release 15 College Station, TX: StataCorp LP 2017). Statistical analysis plans can be obtained from the first author (PKE).

## Discussion

In the current study, we will investigate the clinical effect of 3 g/day of adjunctive NAC treatment on symptoms of bipolar depression utilizing a randomized, double-blinded, parallel group, placebo-controlled design. People diagnosed with bipolar disorder predominantly experience depression, which is currently challenging to treat (Grande et al. 2016). It is evident that a large group of patients do not respond adequately to traditional pharmacotherapy and/or psychotherapy, which creates a need for novel treatment opportunities. If this trial indicates that NAC treatment significantly reduces symptoms of depression, NAC may become an important part of the pharmacotherapeutical options for bipolar disorder.

NAC is well-tolerated with few and often mild adverse events (Fernandes et al. 2016), and is available without prescription in many countries. The selected dosage in this trial has been well-tolerated in previous trials (Prado et al. 2015; Mardikian et al. 2007). NAC is hypothesized to decrease symptoms of depression at least in part due to its antioxidant properties. At present, few studies have investigated the effects of NAC in patients with bipolar depression, and the results have been mixed. Our study therefore will add to the limited clinical database (Berk et al. 2008, 2012).

The main strengths of the trial are the double-blinded design, with only two raters, and the possibility to do the study interviews in the patients’ home, which should reduce drop-outs.

To increase adherence, the study medication is formulated as tablets instead of effervescent tablets, making is possible to take it along with existing medication. To reflect usual clinical practice, all kinds of non-study pharmaceutical treatment are allowed, including changes prior to and after inclusion. However, this may diminish the chances to detect a potential superiority of NAC over placebo.

The novelty of this study is the collection of several biological materials to explore the oxidative balance, inflammation and DNA damage related to the disorder and the treatment. Additionally, the study will shed light on the possible immune modulatory effects of NAC treatment. The markers may provide an insight to the potential mechanisms underlying the pathophysiology of BD.

### Dissemination of results

The results from this study will be presented at national and international conferences and published in peer-reviewed journals.

## Abbreviations

AE: adverse events
ANCOVA: ANalysis of COVAriance
AR: adverse reactions
CGI-S: Clinical Global Impression Scale-Severity scale
CONSORT: The Consolidated Standards of Reporting Trials
CRF: Case Report Form
CTN: Connie T. Nielsen
DSM-IV: Diagnostic and Statistical Manual of Mental Disorders 5
ECT: electroconvulsive therapy
GAF-F: Global Assessment of Functioning Scale
GAF-S: Global Assessment of Symptoms Scale
GCP: good clinical practice
GDP: good distribution practice
GEE: generalized estimating equation
GMP: good manufacturing practice
GSH: glutathione
HEP: Henrik E. Poulsen
HOS: Helle O. Soerensen
ITT: intention to treat
LMERM: linear mixed effects repeated measures
MADRS: Montgomery–Asberg Depression Rating Scale
M.B: Michael Berk
MES: Melancholia Scale
M.I.N.I-interview: Mini International Neuropsychiatric Interview
MNAR: missing nor at random
NAC: *N*-acetylcysteine
OMD: Olivia M. Dean
PKE: Pernille K. Ellegaard
REDCap: research electronics data capture
REN: René E. Nielsen
RWL: Rasmus W. Licht
SAE: serious adverse event
SAR: serious adverse reaction
SPIRIT: The Standard Protocol Items: Recommendations for Intervention Trials
WHO-5: WHO-5 Well-Being Index
YMRS: Young Mania Rating Scale
